# Retraction: Effects of Iron Overload on the Bone Marrow Microenvironment in Mice

**DOI:** 10.1371/journal.pone.0341204

**Published:** 2026-01-20

**Authors:** The PLOS One Editors

Following the publication of this article [1] concerns were raised with the mouse model, the results presented in Figs 1 and 3 and the references. Specifically,

- The iron-overload mouse model used in this study was previously reported by the authors of [1] in [2], which was not cited in [1].
- Figs 1B and 1C appear similar to Figs 1B and 1C in [3], corrected in [4].
- The upper two panels in Fig 1C appear similar to the right two panels in Fig 1 in [2] when the aspect ratio is adjusted.
- The Fig 1B Control Bone marrow panel appears similar to the Fig 4C Control panel in [5] when rotated 90°.
- The Fig 1B Fe Bone marrow panel appears similar to the Fig 4C High dose panel in [5] when rotated 90°.
- The upper left panel in Fig 1C appears similar to the Fig 4D Low dose panel in [5] when rotated 90° with aspect ratio adjusted.
- The Fig 3A Control and Fe+DFX panels appear to overlap at different magnifications.
- The Fig 3A Fe and Fe+NAC panels appear to overlap at different magnifications.
- The Fig 3B Fe and Fe+NAC panels appear to overlap at different magnifications.
- References 10 [6] and 33 [8] were retracted prior to the publication of [1] in [7] and [9] respectively.

The corresponding author stated that Fig 1A in [1] presents the labile iron pool level (LIP) of bone marrow derived mesenchymal stem cells (BM-MSCs), and Fig 1A in [3], corrected in [4] presents the LIP of bone marrow mononuclear cells (BMMNCs). Regarding the similarities between Figs 1B and C in [1] and in [3], corrected in [4], the corresponding author stated that [1] focuses on the effect of bone marrow microenvironment by iron overload, and [3], corrected in [4], focuses on the hematopoiesis of bone marrow by iron overload, and that [1] and [3], corrected in [4], share the same iron overload mouse model from experiments carried out at the same time. They stated that they established the iron overload mouse model once, using the same Figs 1B and C in [1] and [3], corrected in [4].

The authors did not respond to editorial requests for comments on the remainder of the concerns.

In light of the above unresolved concerns which call into question the reliability and integrity of the published results, the *PLOS One* Editors retract this article.

YZ, WZ, DL, XChai, XCao, JMeng, JC, XX, QL, JMu, JS, and AM either did not respond directly or could not be reached. MZ did not respond to the final editorial decision.

The upper two panels in Fig 1C report material similar to that published in Fig 1 in [2], published in 2014 by Zhongguo Yixue Kexueyuan. The upper two panels in Fig 1C are not offered under a CC BY license and are therefore excluded from this article’s [1] license.

Since the publication of [1], Reference 6 [10,11] has been retracted [12].
